# Effects from RF spoiling disequilibrium in the background offsets of phase-contrast velocity imaging

**DOI:** 10.1186/1532-429X-14-S1-W56

**Published:** 2012-02-01

**Authors:** Peter D Gatehouse, Andreas Greiser, David N Firmin

**Affiliations:** 1 Royal Brompton Hospital, London, UK; 2 Siemens Medical Systems, Erlangen, Germany

## Summary

To investigate how disequilibrium of spoiling affects background offset errors in phase-contrast velocity images.

## Background

Phase-contrast imaging normally uses gradient-echoes with RF spoiling and phase-encode rewinding with a fixed dephasing by gradient “spoiler” pulses in each TR (time between RF pulses) studied in detail (1) where effective artifact suppression in magnitude images required >≈ 8PI dephasing over the slice thickness, with >≈ 2PI dephasing over an FE pixel. However, the effectiveness of spoiling depends on establishing a steady-state. In phase-contrast imaging, background offsets are known to stabilise in continuous scanning such as retrospective gating, but sequence interruptions can be necessary eg in navigator-gating or slice-tracking. Unsteady background offsets are generally ascribed to disturbed equilibrium in eddy-current effects. This abstract is a first investigation of contributions from disequilibrium in spoiling.

## Methods

To separate the two sources of unsteady background, the RF pulses in a non-segmented prospectively-triggered 50-frame cine phase-contrast 1m/s sequence were disabled in frames 21-30 while the gradient waveforms continued for those frames. Therefore data from frames 1-20 (“pre” RF interruption) include both sources while frames 31-50 (“post”) contain only spoiling disequilibrium effects. Background velocity offsets in muscle and fat were measured at 3T as functions of flip angle (20 - 30°), spoiler gradients (50,100,150% of the values in ref. 1 ) and TR (4.2 - 13.1ms).

## Results and discussion

**Figure 1:**
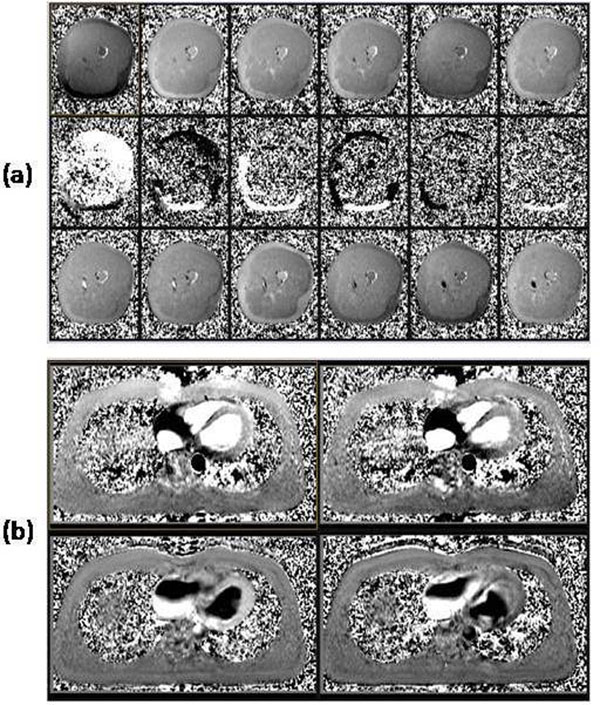
Phase-contrast velocity images all displayed at same window and level settings. (a) Top row: Cine frames at start of the prospectively triggered cine. Middle row: During the frames with RF disabled but continuing gradient activity. Bottom row: Frame 31 onward with RF re-enabled. (b) Top row is two cine frames of a triggered cine acquisition at one raw data line per cardiac cycle per frame (“non-segmented”), where fat and muscle exhibit different variations. The bottom row shows that acquiring multiple phase-encode lines per cardiac cycle largely transmutes background instability into phase-encode ghosting.

The different muscle and fat variations (Figure 1) imply incomplete spoiling, since eddy currents would affect both equally. Furthermore, the variations also occur post RF interruption. The results in Table 1 show: stronger variations in fat than muscle ; pre variations slightly larger than post ; no impact of greater spoiler amplitude; variations decrease sharply with lower flip angle and decrease with longer TR. A similar effect was observed in cardiac imaging (Figure 1b). Averaging was not used for the results, but was used in Figure 1 to display the effect clearly. Further work is needed to determine if the transition to equilibrium for RF spoiling can be optimised for phase-contrast imaging, and also what impact this effect has on flow measurements and whether it also occurs in blood. Thorough investigation of the optimum flip angle in phase-contrast imaging is also indicated.

**Table 1:** Temporal stdev (cm/s) of tissue ROIs

	Pre	Pre	Post	Post
Conditions	Muscle	Fat	Muscle	Fat
FA30,SP150%	0.51	1.29	0.26	0.87
FA30,SP100%	0.47	1.3	0.27	0.87
FA30,SP50%	0.47	1.34	0.26	0.85
FA20,SP150%	0.25	0.64	0.18	0.51
FA20,SP100%	0.26	0.63	0.17	0.5
FA20,SP50%	0.23	0.67	0.22	0.5
TR4.2ms	0.35	0.56	0.29	0.48
TR5.6ms	0.27	0.61	0.17	0.48
TR8.2ms	0.21	0.48	0.22	0.43
TR10.7ms	0.2	0.22	0.2	0.41
TR13.2ms	0.2	0.26	0.13	0.38

Pre=Temporal stdev over Frames 1-20. Post=Temporal stdev over Frames 31-50. Frames 21-30 had no RF but continued gradients. The FlipAngle and SpoilerAmplitude tests were all at TR5.6ms. The TR tests were all at FA20 SP100%.The temporal stdev noise level in muscle was approx 0.2cm/s.

## Conclusions

Most of the results are consistent with incomplete spoiling in the early frames of a cine as the spoiling equilibrium is established, for example the longer T2 of fat explains its larger signals in higher-order pathways, which are reduced by lower flip angle. This effect stabilizes after a longer series of continuous gradient and RF activity, and only marginally exceeds random noise in muscle at lower flip-angles. However, for some phase-contrast sequences this effect may be significant in the background offset.

## Funding

NIHR Cardiovascular Biomedical Research Unit funding.
